# Increased Urinary Extracellular Vesicles and Reduced Expression of NEDD4L in Patients With Type 2 Diabetes and Diabetic Nephropathy

**DOI:** 10.1155/jdr/3850490

**Published:** 2026-03-09

**Authors:** Nikhil Thyagarajan, Richard Le Leu, Sharad Kumar, Shilpanjali Jesudason, Jantina A. Manning

**Affiliations:** ^1^ Central Northern Adelaide Renal and Transplantation Service (CNARTS), Central Adelaide Local Health Network, Royal Adelaide Hospital, Adelaide, South Australia, Australia, rah.sa.gov.au; ^2^ Adelaide Medical School, Faculty of Health and Medical Sciences, The University of Adelaide, Adelaide, South Australia, Australia, adelaide.edu.au; ^3^ Centre for Cancer Biology, University of South Australia and SA Pathology, Adelaide, South Australia, Australia, centreforcancerbiology.org.au; ^4^ Centre for Cancer Biology, Adelaide University, Adelaide, South Australia, Australia, adelaide.edu.au

**Keywords:** diabetic nephropathy, extracellular vesicles, kidney biopsy, protein expression, tubular injury, urine

## Abstract

**Introduction:**

Diabetic nephropathy (DN) is a leading cause of chronic kidney disease. Current diagnosis requires the persistent presence of albuminuria and/or reduction in estimated glomerular filtration rate, often detected only at late stages of disease. In recent years, extracellular vesicles (EVs), lipid bilayer membrane–enclosed particles that shed from cells, have gained momentum as a potential source of biomarkers for DN, reflecting pathological changes in this condition. Our previous work has demonstrated robust expression of the NEDD4L ubiquitin ligase in urinary EVs, with the loss of this protein in mice resulting in kidney disease. Reduced NEDD4L expression has been reported in the kidneys of patients with DN. Hence, in this study we analysed NEDD4L expression in kidney biopsies from patients with Type 2 diabetes mellitus (T2DM) and DN, as well as the release of urinary EVs from patients with DN and control subjects without diabetes. We assessed whether the level of NEDD4L in these vesicles reflects NEDD4L expression in kidney biopsy samples.

**Methods:**

Kidney biopsies (slides of control healthy kidney tissue taken adjacent to a suspected tumour site from five patients who had performed biopsy for suspicion of oncological pathology, and slides of kidney tissue from five patients with T2DM and DN) were utilised. Urine samples (from 21 control participants without diabetes, and from 21 patients with T2DM and DN) were collected. EVs were isolated and characterised from urine samples. NEDD4L protein expression levels were assessed in kidney biopsies and urinary EV samples.

**Results:**

Urine samples from patients with DN exhibited a heterogeneous population of EVs, with increased EV numbers and protein concentrations compared with controls without diabetes. Within EVs, NEDD4L protein expression was significantly reduced in patients with DN as compared with controls without diabetes.

**Conclusion:**

We reported a higher concentration of a heterogeneous population of urinary EVs in patients with T2DM and DN, with decreased NEDD4L protein levels that reflect the findings observed in the kidney biopsy samples of such patients. These findings support the potential use of urinary EVs as biomarkers of T2DM‐related DN and suggest that lower NEDD4L levels within these vesicles may be an indicator of the extent of DN‐associated tubular injury.

## 1. Background

Diabetic nephropathy (DN) develops in up to 40% of people with diabetes mellitus [1, 2] and is the leading cause of kidney failure requiring renal replacement therapy, accounting for 36% of incident cases in Australia and 46% in Aotearoa (New Zealand) [3] and approximately 30% of cases worldwide [3–6]. In addition to vascular and glomerular injuries, loss of tubular epithelial cells through programmed cell death (apoptosis) contributes extensively to this disease [7]. Current screening for DN relies on monitoring for micro‐ or macroalbuminuria and reduction in estimated glomerular filtration rate (eGFR), the latter being a late sign of disease often not detected until there is already significant kidney cell death and kidney injury [8]. Hence, strategies for early disease detection (including the identification of novel biomarkers) and effective interventions to delay disease progression are of critical importance to improve clinical outcomes of DN patients.

Extracellular vesicles (EVs) are lipid bilayer membrane–enclosed particles that contain bioactive molecules from their cell of origin [9], including from diabetic kidneys [10]. They are released by almost every cell, but are of particular interest as both a therapeutic target and a non‐invasive means of biomarker measurement in the genitourinary tract [9, 11]. Kidney‐derived urinary EVs can mediate crosstalk between glomerular and tubular cells, initiate communication between different segments of the nephron and can transfer biological material such as RNAs, lipids and proteins [11]. As non‐invasive biomarkers, urinary EVs have been investigated in rodent models of acute kidney injury (AKI), as well as in podocytopathies (in vitro and in vivo studies), Type 1 diabetes and kidney transplant recipients (for the assessment of kidney allograft function) in humans [11, 12]. Recently, it has emerged that the content of urinary EVs, particularly proteins and RNA, can reflect pathological changes in DN [10, 13]. The content of EVs varies, depending on the health or disease state of their parental cell [13]. However, we have previously detected robust expression of the ubiquitin ligase NEDD4L (neural precursor cell expressed developmentally downregulated 4‐like) in EVs isolated from human urine samples (urinary EVs obtained from pregnant women with and without pre‐eclampsia) [14].

Our earlier work demonstrated that NEDD4‐2 (neural precursor cell expressed developmentally downregulated 4‐2) [known as NEDD4L in humans] is important in maintaining a healthy kidney in mice [15]. NEDD4L is a HECT (homologous to the E6‐AP C terminus) type of ubiquitin ligase that transfers ubiquitin onto substrates [16]. Many of the NEDD4L substrates are plasma membrane proteins that, upon ubiquitination, undergo degradation or recycling, thus modulating cell signalling pathways and functional outcomes, such as ion and nutrient transport, cellular growth responses and tissue‐level homeostasis [16]. NEDD4L regulates several key ion channels and transporters implicated in the pathophysiology of kidney disease, including the epithelial sodium channel (ENaC) [17], sodium–glucose cotransporter‐1 (SGLT1) [18] and sodium–chloride cotransporter (NCC) [17, 19]. Renal tubule–specific knockout of NEDD4‐2 in mice results in gradual, salt‐sensitive kidney damage, similar to chronic kidney disease (CKD) characterised by elevated ENaC levels, fibrosis, apoptosis and tubular damage [20–22]. Importantly, NEDD4L/NEDD4‐2 gene is highly conserved between mice and humans, in terms of both sequence and function [16]. Recently, NEDD4L variants have been associated with Type 2 diabetes mellitus (T2DM) [23] and reduced NEDD4L expression has been observed in the kidneys of patients with DN, correlating with aberrant electrolyte transport [24]. Recent work has also identified reduced NEDD4‐2 expression in the kidneys of diabetic mice [25].

NEDD4‐2 and its adaptors are also implicated in the regulation of EV biogenesis [26], with NEDD4‐2 ubiquitin ligase being released in EVs, including urinary EVs [14]. In this study, we isolated urinary EVs from patients with T2DM and comorbid DN and from control subjects without diabetes, measuring EV concentration and size, as well as EV protein concentration. NEDD4L expression in both kidney biopsies and urinary EVs was assessed to determine whether levels of this protein may reflect pathological changes in DN patients.

## 2. Materials and Methods

### 2.1. Ethics Approval

Ethics approval to access previously stored human kidney biopsy samples from SA Pathology (Government of South Australia) was approved by the Central Adelaide Local Health Network Human Research Ethics Committee (CALHN HREC) (Approval Code #13208; date of approval: 12 June 2020). Urine and clinical data collection was approved by the CALHN HREC (Approval Code 18347; date of approval: 12 October 2023) and University of South Australia HREC (Approval Code #205882; date of approval: 12 October 2023), with written informed consent obtained.

### 2.2. Kidney Biopsy Sample Collection

Paraffin‐embedded slides from five patients with T2DM who underwent kidney biopsy and histological confirmation of DN and paraffin‐embedded slides of control healthy kidney tissue taken adjacent to a suspected tumour site from five patients who had performed kidney biopsy for suspicion of oncological pathology were obtained from pathologists at the Royal Adelaide Hospital. The paraffin‐embedded slides from subjects who had previously performed kidney biopsy for diagnostic purposes (histological confirmation of DN or suspicion of oncological pathology) were prospectively collected and analysed.

### 2.3. Immunostaining of Kidney Biopsies

Paraffin sections were deparaffinized and hydrated in a graded ethanol series. Heat‐mediated antigen retrieval was conducted by boiling for 10 min in 10‐mM citric acid (pH 6). Tissue sections were blocked with 10% goat serum and then incubated with anti‐NEDD4‐2 antibody (in house [27]; 1:100) overnight at 4°C and then rhodamine‐labelled DBA (Vector Laboratories, Newark, California, United States) and Alexa Fluor 488 (Thermo Fisher, Waltham, Massachusetts, United States) for 3 h at room temperature. Sections were counterstained with Hoechst 33342 (Thermo Fisher, Waltham, Massachusetts, United States) and mounted in ProLong Gold Antifade reagent (Thermo Fisher, Waltham, Massachusetts, United States). Stained samples were imaged using a LSM 800 confocal microscope using Zen 2011 (Black Edition) Version 8.1.5.484 (Carl Zeiss Microscopy, Jena, Germany). Image analysis was conducted using Zen 2011 (Blue Edition, Carl Zeiss Microscopy, Jena, Germany) and Adobe Photoshop (Version 26.11.2, Adobe Inc., San Jose, California, United States). Quantitation of NEDD4L fluorescence intensity was conducted in ImageJ (NIH), with the total fluorescence intensity divided by the cell number for each tubule to normalise for size differences.

### 2.4. Participant Recruitment and Clinical Data Collection for Patients With T2DM and DN

This was a prospective observational study involving a clinical lead nephrologist, a clinical team member and scientist. This study involved collecting baseline clinical characteristics and spot urine samples of up to 50 mL from patients with T2DM and DN, and from control participants without diabetes. Specific baseline clinical characteristics included age, sex, weight, self‐reported height (for reasons of feasibility in the outpatient clinic setting), body mass index (BMI), blood pressure, glycated haemoglobin (HbA1c), urinary albumin‐to‐creatinine ratio (uACR), eGFR, duration of diabetes, medical comorbidities, and use of medications.

Participants with DN (*n* = 21) were recruited purposively from the adult nephrology outpatient population at the Royal Adelaide Hospital between 6 December 2023 and 29 May 2024. Participants were included in the study if they had a known history of T2DM, uACR ≥ 3 mg/mmol, CKD Stage G2–4 (eGFR 15–60 mL/min/1.73 m^2^) as assessed by eGFR values over at least 3 months (calculated using the 2021 CKD‐EPI equation) [28–30], and a clinical or biopsy diagnosis of DN. CKD was defined as abnormalities of kidney structure or function, present for a minimum of 3 months, with implications for health [28]. A clinical diagnosis of DN was made in the setting of T2DM and CKD [28]. Exclusion criteria included an age less than 18 years, pregnancy, diabetes types other than T2DM, CKD Stage G5 or other significant confounding medical conditions contributing to renal impairment, with the exception of hypertension.

Baseline anthropometrics (age, sex, weight, self‐reported height [for reasons of feasibility in the outpatient clinic setting] and BMI) and blood pressure were collected. Medication lists of all participants were examined from either self‐reporting or electronic medical records (EMRs), and the use of antihypertensive drugs, sodium–glucose cotransporter 2 (SGLT2) inhibitors, other oral glucose–lowering medications, insulin or other relevant medications was recorded. The presence of known hypertension was recorded for all participants. Other relevant comorbidities and the most recent creatinine, eGFR and uACR values were recorded from the EMRs.

### 2.5. Participant Recruitment and Clinical Data Collection for Control Participants Without Diabetes

Control participants without any diagnosis or indication of diabetes and kidney disease (*n* = 21) were selected prospectively by convenience sampling from staff, research students and medical trainees, or contacts approached by investigators within the Royal Adelaide Hospital and Centre for Cancer Biology (University of South Australia and SA Pathology) after recruitment flyers and study information were disseminated. Inclusion criteria included an age above 18 years, ability and willingness to provide informed consent for study participation, no prior diagnosis of diabetes and no history of kidney disease. The recruitment period was from 6 February 2024 to 15 April 2024. Exclusion criteria included other medical comorbidities outside of mild‐to‐moderate hypertension (absence of resistant hypertension, severe hypertension and hypertension‐mediated end‐organ damage) [31], use of medications other than antihypertensive drugs and pregnancy. Baseline anthropometrics (age, sex, weight, self‐reported height and BMI) were collected. Blood pressure was measured, and the use of antihypertensive drugs was recorded. Baseline assessment of laboratory biochemical parameters (including eGFR) was not performed.

### 2.6. Urine Collection

Up to 50 mL of urine was collected from each recruited participant into a 70‐mL sterile specimen jar and immediately placed on ice for 0–4 h before being aliquoted and frozen at −80°C.

### 2.7. Urinary Creatinine Measurement

A total of 100 *μ*L of aliquoted urine frozen at −80°C were thawed, vortexed and centrifuged at 2300 g to remove debris. A 1:20 dilution of urine supernatant was used in the Creatinine Urinary Detection Kit (Invitrogen, #EIACUN, Thermo Fisher, Waltham, Massachusetts, United States) as per manufacturer′s instructions.

### 2.8. EV Isolation From Urine

A total of 3 mL of aliquoted urine was thawed and vortexed, then centrifuged at 1600 g at 4°C for 10 min to remove cellular debris. The supernatant was transferred to polycarbonate tubes and centrifuged at 17000 g at 4°C for 15 min (Optima TLX 120 CE ultracentrifuge, Beckman Coulter, Brea, California, United States). The supernatant was transferred into new tubes and ultracentrifuged at 100000 g at 4°C for 2 h to pellet EVs. The supernatant was discarded and the pellet resuspended in 35 *μ*L of Verhagen lysis buffer (150 mM NaCl, 20 mM Tris‐HCl pH 7.5, 2 mM EDTA, 10% glycerol and 1% Triton X‐100) for immunoblotting, 20 *μ*L of filtered phosphate‐buffered saline (PBS) for transmission electron microscopy, or 1 mL of PBS for nanoparticle tracking analysis.

### 2.9. Transmission Electron Microscopy (TEM)

A total of 5 *μ*L of sample was loaded onto a prepared copper mesh formvar grid (ProSciTech, Kirwan, Queensland, Australia). Liquid was blotted off and sample fixed with 4% paraformaldehyde for 2 min, then washed with distilled H_2_O, and counterstained with 0.5% uranyl acetate for 30 s. Samples were observed and images captured using the Tecnai G2 Spirit TEM (FEI Company, Hillsboro, Oregon, United States) at the University of Adelaide Microscopy Facility.

### 2.10. Nanoparticle Tracking Analysis (NTA)

1:10 dilution of EV sample was resuspended in 1 mL of PBS and processed with a NanoSight NS300 (Malvern Panalytical, Malvern, United Kingdom). Five captures were recorded for each sample and averaged. Nanoparticle number and size distribution were calculated for seven control samples and for seven DN samples.

### 2.11. Immunoblotting

A total of 15 *μ*L of each EV sample was loaded onto 4%–20% precast stain‐free polyacrylamide gels (Bio‐Rad Laboratories, Hercules, California, United States) for separation by electrophoresis. Proteins were activated by 5 min of UV exposure, then transferred to polyvinylidene difluoride (PVDF) membrane using a Trans‐Blot Turbo Transfer System (Bio‐Rad Laboratories, Hercules, California, United States). Total protein amount was captured using a GelDoc Go Gel Imaging System (Bio‐Rad Laboratories, Hercules, California, United States). Membranes were then blocked with 5% skim milk and immunoblotted with primary antibody diluted in 5% skim milk in TBS (Tris‐buffered saline with 0.5% Tween 20) incubated overnight at 4°C. Antibodies all at 1:1000 were anti‐NEDD4‐2 antibody (in house [27]), which also detects NEDD4L [14]; anti‐CD81 (#D3N2D, Cell Signaling Technology, Danvers, Massachusetts, United States); anti‐Annexin A1 (#214486, Abcam, Cambridge, United Kingdom); anti‐TSG101 (#ab83, Abcam, Cambridge, United Kingdom); anti‐GM130 (#610823, BD Transduction Laboratories, Franklin Lakes, New Jersey, United States); anti‐THP (#8595‐0054, AbD Serotec, Bio‐Rad Laboratories, Hercules, California, United States) followed by incubation with horseradish peroxidase (HRP) secondary antibody (Bio‐Rad Laboratories, Hercules, California, United States). HRP signals were visualized by using West Femto (Thermo Fisher, Waltham, Massachusetts) on a ChemiDoc MP Imaging System (Bio‐Rad Laboratories, Hercules, California, United States). Protein quantitation was conducted using Image Lab 6.1 Software (Bio‐Rad Laboratories, Hercules, California, United States). For total proteins, the adjusted volume of the entire lane was calculated. For individual proteins, the band was selected and volume calculated. Background subtraction was set at 21.2‐mm disc size for all lanes. Levels were normalised to a standard sample that was loaded on all gels. NEDD4L levels were normalised to the EV biomarkers Annexin A1 as a plasma membrane protein, and CD81 and TSG101 as commonly used urinary exosome markers [32].

### 2.12. Statistical Analysis

For quantitation of biopsy NEDD4L fluorescence and EV particle analysis, an unpaired Student′s *t*‐test was utilised. For remaining data, power calculations were conducted prospectively based on an unpaired analysis of variance (ANOVA) comparison of means, assuming a standard deviation of 30%, and 30% difference in means. The significance level (*α*) was set at 0.05, determining that a total sample size of 42 participants (21 participants per group) was collected and all samples were used, with no exclusions. Baseline clinical characteristics were reported with summary statistics. Correlation coefficients for parametric data were calculated using Pearson′s correlation coefficient, with the significance level (*α*) set at 0.05. Normality was assumed by the central limit theorem. For comparison between two groups where normality criteria were not satisfied, the Mann–Whitney *U* test was selected as a rank sum comparison of medians in two unpaired groups. The significance level (*α*) was set at 0.05. The statistical software used were IBM SPSS (Version 29.0.1.0) and GraphPad Prism (Version 10.2.3; GraphPad Software). Results were rounded to two decimal places, with *p* values rounded to three decimal places.

## 3. Results

### 3.1. Reduced NEDD4L Expression in Kidney Biopsies From DN Patients

NEDD4L expression was assessed by immunofluorescence staining in kidney biopsy samples from patients with T2DM and DN and in kidney biopsy samples consisting of slides of control healthy kidney tissue taken adjacent to a suspected tumour site from five patients who had performed kidney biopsy for suspicion of oncological pathology. In control healthy kidney tissues, NEDD4L had intense expression in a distinct subset of renal tubules (Figure 1a,b). NEDD4L expression was colocalised with a subset of cells displaying weak expression of the renal collecting duct marker *Dolichos biflorus* agglutinin (DBA), indicating collecting duct localisation [33, 34]. There was a marked reduction of NEDD4L staining in the renal tubules of patients with DN (mean intensity: 0.39 AU) as compared with control kidney tissues without DN (mean intensity: 1.00 AU), and this was statistically significant when quantitated over kidney biopsy samples from five DN patients and healthy kidney tissues from five patients who had performed kidney biopsy for suspicion of oncological pathology (*p* < 0.001, Figure 1c).

Figure 1Reduced NEDD4L expression evidenced by immunofluorescence staining in kidney biopsies from patients with Type 2 diabetes mellitus (T2DM) and diabetic nephropathy (DN). (a) Paraffin‐embedded kidney biopsy samples consisting of control healthy kidney tissues adjacent to a suspected tumour site (from five patients who had performed kidney biopsy for suspicion of oncological pathology) and paraffin‐embedded kidney biopsy samples from five patients with T2DM and DN (taken for histological confirmation) were stained with NEDD4L (green), the collecting duct marker *Dolichos biflorus* agglutinin (DBA, red)) and the Hoechst 33342 DNA stain (blue). Three representative images are shown for both control and DN biopsies, with a total of five biopsy slides from control and five biopsy slides from patients with T2DM and DN analysed. (b) Higher magnification of biopsy samples. (c) NEDD4L intensity was measured and divided by cell number for each tubule. Renal tubules in five fields of view were measured for *n* = 5 different patient samples in each group, revealing a decrease in NEDD4L expression in DN samples. Data are presented as *m* *e* *a* *n* ± *s* *t* *a* *n* *d* *a* *r* *d* *e* *r* *r* *o* *r* *of* *the* *m* *e* *a* *n* (SEM) and statistical significance determined using an unpaired two‐tailed Student′s *t*‐test. Abbreviations: AU, arbitrary units.  ^∗∗∗^ *p* < 0.001.

**Figure:**
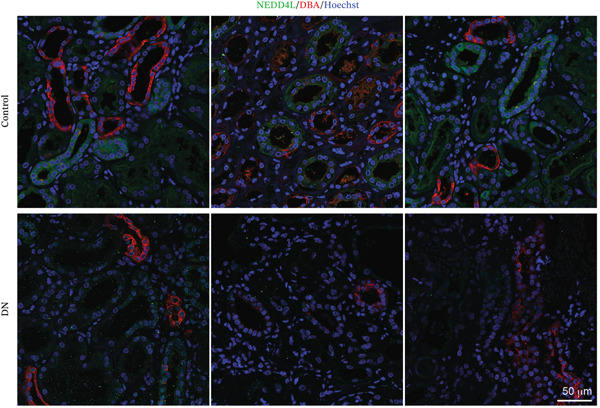
(a)

**Figure:**
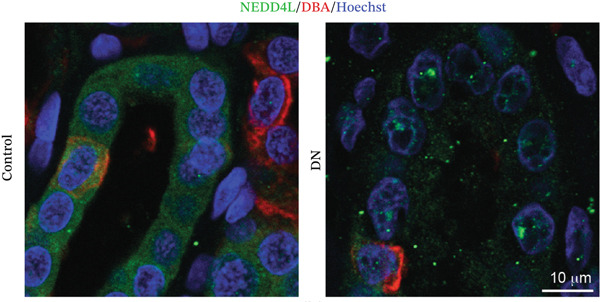
(b)

**Figure:**
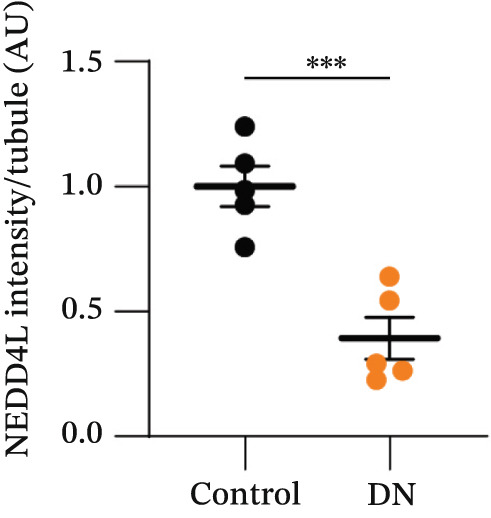
(c)

### 3.2. Increased Numbers of Urinary EVs in DN Patients

To assess whether the reduction of NEDD4L expression in DN patients could also be observed in urine samples from DN patients, we collected urine from controls without diabetes and from patients with DN (*n* = 21 participants for each group), with baseline clinical characteristics of study participants summarised in Table 1. Statistical differences in baseline characteristics between the study groups were not calculated as this pilot study with a small sample size was not adequately powered for subgroup analysis.

**Table 1:** Baseline clinical characteristics of the study participants.

	Control subjects without diabetes (*n* = 21)	Patients with T2DM and DN (*n* = 21)
Age (years) (median, IQR)	51 (36.0)	73 (16.0)
Male sex (*n*, %)	10 (47.6%)	17 (81.0%)
Female sex (*n*, %)	11 (52.4%)	4 (19.0%)
Weight (kg) (median, IQR)	71.0 (17.0)	85.5 (23.3)
Height^a^ (m) (median, IQR)	1.7 (0.1)	1.7 (0.1)
BMI (kg/m^2^) (median, IQR)	24.1 (3.6)	28.8 (8.5)
Proportion of subjects with underweight (BMI: < 18.5 kg/m^2^) (*n*, %)	0 (0%)	0 (0%)
Proportion of subjects with normal weight (BMI: 18.5–24.9 kg/m^2^) (*n*, %)	13 (61.90%)	4 (19.00%)
Proportion of subjects with overweight (BMI: 25.0–29.9 kg/m^2^) (*n*, %)	6 (28.57%)	7 (33.33%)
Proportion of subjects with class 1 obesity (BMI: 30.0–34.9 kg/m^2^) (*n*, %)	2 (9.53%)	6 (28.67%)
Proportion of subjects with Class 2 obesity (BMI: 35.0–39.9 kg/m^2^) (*n*, %)	0 (0%)	4 (19.00%)
Proportion of subjects with Class 3 obesity (BMI: ≥ 40.0 kg/m^2^) (*n*, %)	0 (0%)	0 (%)
Systolic BP (mmHg) (median, IQR)	126 (13.5)	130 (19.5)
Diastolic BP (mmHg) (median, IQR)	80 (13.0)	71 (17.3)
Duration of diabetes (years) (median, IQR)	Not applicable	25 (18.8)
HbA1c (%) (median, IQR)	N/A	7.7 (2.2)
Serum creatinine (*μ*mol/L) (median, IQR)	N/A	185.5 (99.3)
EGFR (mL/min/1.73 m^2^)^b^	N/A	28 (23.3)
UACR (mg/mmol) (median, IQR)	N/A	28.9 (133.6)
Prevalence of hypertension (*n*, %)	6 (28.6%)	16 (76.2%)
Proportion of subjects taking a RAAS blocker (*n*, %)	6 (28.6%)	16 (76.2%)
Proportion of subjects taking SGLT2 inhibitors (*n*, %)	0 (0%)	8 (38.1%)
Proportion of subjects taking thiazolidinediones (*n*, %)	0 (0%)	0 (0%)
Proportion of subjects taking metformin (*n*, %)	0 (0%)	12 (57.0%)
Proportion of subjects taking insulin (*n*, %)	0 (0%)	2 (9.52%)
Proportion of subjects taking GLP‐1 receptor agonists (*n*, %)	0 (0%)	3 (14.29%)
Proportion of subjects taking DPP‐4 inhibitors (*n*, %)	0 (0%)	4 (19.00%)
Proportion of subjects taking sulfonylureas (*n*, %)	0 (0%)	2 (9.52%)
Proportion of subjects taking alpha‐glucosidase inhibitors (*n*, %)	0 (0%)	1 (4.76%)

Abbreviations: BMI, body mass index; BP, blood pressure; DN, diabetic nephropathy; DPP‐4, dipeptidyl peptidase‐4; eGFR, estimated glomerular filtration rate; GLP‐1, glucagon‐like peptide‐1; HbA1c, glycated haemoglobin; IQR, interquartile range; N/A, not available; RAAS, renin‐angiotensin‐aldosterone system; SGLT2, sodium‐glucose cotransporter 2; T2DM, Type 2 diabetes mellitus; uACR, urinary albumin‐to‐creatinine ratio.

^a^Height was self‐reported by participants for reasons of feasibility in the outpatient clinic setting.

^b^eGFR was calculated using the 2021 CKD‐EPI (Chronic Kidney Disease Epidemiology Collaboration) equation.

The approach for urine analysis is shown in Figure 2a. Spot urine creatinine concentration was not significantly different between control and DN patient samples (Figure S1).

Figure 2Isolation and quantification of urinary extracellular vesicles (EVs). (a) Experimental approach. (b) Transmission electron microscopy (TEM) images of EVs isolated from control participants without diabetes; higher magnification image details sizes of some EV nanoparticles. (c) Particle analysis (by NTA, nanoparticle tracking analysis) reveals an increased concentration of EVs in DN urine samples, normalised to urinary creatinine levels (urinary particle concentrations were normalised to urinary creatinine concentration [mg/dL]). (d) Particle size distribution was similar between the two study groups, as identified by NTA. (e) Particle size distribution showed a significant increase in the number of 100–200‐nm particles in DN samples. Data (for *n* = 7 for each group) are presented as *m* *e* *a* *n* ± *s* *t* *a* *n* *d* *a* *r* *d* *e* *r* *r* *o* *r* *of* *the* *m* *e* *a* *n* (SEM), and statistical significance were determined using an unpaired two‐tailed Student′s *t*‐test.  ^∗^ *p* < 0.05;  ^∗∗∗^ *p* < 0.001. Figure 2a was created with http://BioRender.com under a CC BY 4.0 license (Kumar 2025; URL: https://BioRender.com/iyxmqkv).

**Figure:**
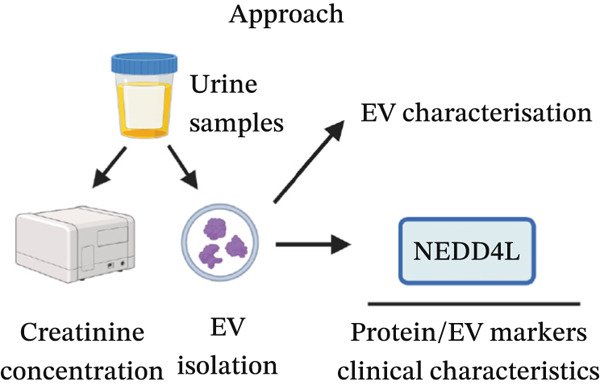
(a)

**Figure:**
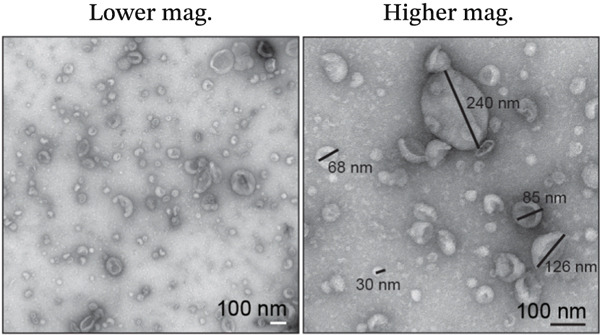
(b)

**Figure:**
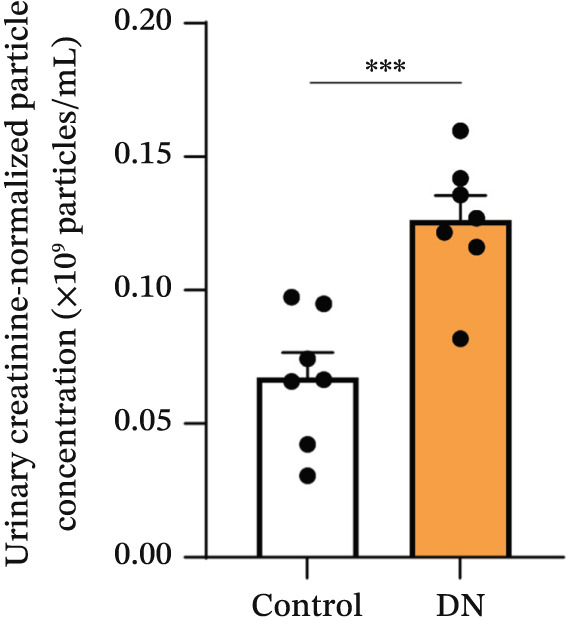
(c)

**Figure:**
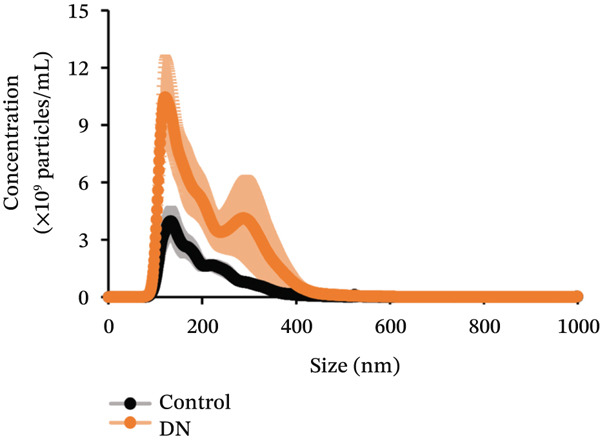
(d)

**Figure:**
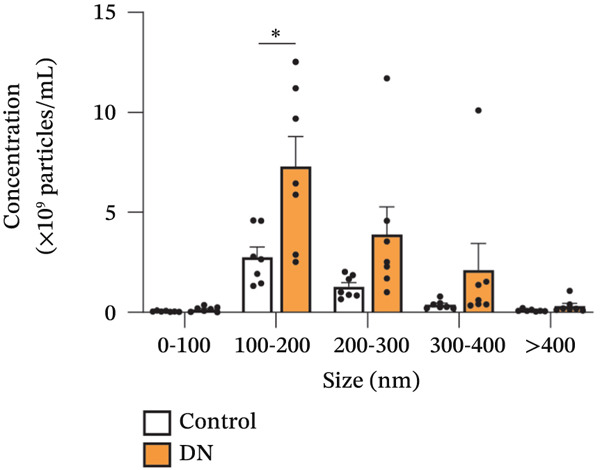
(e)

Imaging of heterogeneous EVs by TEM demonstrated double membrane–enclosed vesicles predominantly in the size range of 30–300 nm, without the presence of contaminating material (Figure 2b). Analysis of a randomly chosen subset of samples (*n* = 7 for each group) by NTA revealed a significant increase (*p* < 0.001) in the number of EVs in the urine of DN patients (mean: 0.13x10^9^ particles/mL, normalised to urinary creatinine) as compared with controls without diabetes (mean: 0.07x10^9^ particles/mL, normalised to urinary creatinine) (Figure 2c). Particle size ranged between 80 and 400 nm (Figure 2d), with the predominant peak around 100 nm. EVs from control and DN patients had a similar size distribution. However, when particles were classified by size, the increase in the number of EVs in DN samples was significant only within the 100–200 nm particle size range, suggesting that these particles were primarily exosomes (*p* < 0.05) (Figure 2e).

### 3.3. Increased Urinary Protein and EV Marker Expression in DN Patients

Protein expression within heterogeneous urinary EVs was determined by immunoblotting, with a representative immunoblot shown in Figure 3a (Sample Set 1). Sample Sets 2 and 3 are shown in Figure S2, whereas complete uncropped immunoblots are shown in Figure S3. Stain‐free blot development and densitometric analysis revealed a significant increase in total protein levels in DN samples (*p* = 0.017; median: 1.91; interquartile range [IQR]: 0.54) as compared with controls (median: 1.01; IQR: 1.93) (Figure 3a,b). Tamm–Horsfall protein (THP) has been reported as a contaminating factor that can hinder EV isolation [35]. However, there was no significant difference in THP density between groups when normalised to the standard (*p* = 0.052; DN median: 0.79; IQR: 0.35; control median: 0.70; IQR: 0.23) or when normalised to total protein levels (*p* = 0.052; DN median: 0.40; IQR: 0.37; control median: 0.68; IQR: 0.46) (Figure S4a,b). There was no expression of the mitochondrial marker GM130, confirming the lack of cellular material in the EV preparations (Figure 3a). Immunoblotting for various EV markers confirmed the increased EV concentration in DN, with higher levels of exosome markers CD81 (*p* < 0.001; median: 1.92; IQR: 1.15) compared with controls (median: 0.95; IQR: 1.82) and higher levels of TSG101 (*p* = 0.009; median: 0.45; IQR: 0.25) compared with controls (median: 0.19; IQR: 0.56), but with no significant difference in the levels of the plasma membrane EV marker Annexin A1 (*p* = 0.114; median: 2.50; IQR: 1.56) compared with controls (median: 1.20; IQR: 3.82) (Figure 3c). After normalising to total protein levels, CD81, TSG101 and Annexin A1 levels were similar in control and DN samples, suggesting that the higher exosome marker levels in DN are due to increased protein and EV content (Figure S4c).

Figure 3Representative immunoblots of isolated EV protein from control and DN urine samples. (a) Membrane was assessed for total protein levels, and then probed for specific proteins. All participant samples across three membranes were normalised to a standard in Lane 1. (b) Total protein levels were significantly increased in DN samples. (c) EV markers CD81 and TSG101, but not Annexin A1, were significantly increased in DN samples as compared with control samples. (d) NEDD4L normalised to total protein or CD81 levels were significantly decreased in DN samples as compared with control samples, but not when they were normalised to TSG101 levels. Data from *n* = 21 are presented as median with interquartile range (IQR). Statistical significance determined using the Mann–Whitney *U* test, *n* = 21.  ^∗^ *p* < 0.05;  ^∗∗^ *p* < 0.01;  ^∗∗∗^ *p* < 0.001. Abbreviations: Rel. pixel density, relative pixel density.

**Figure:**
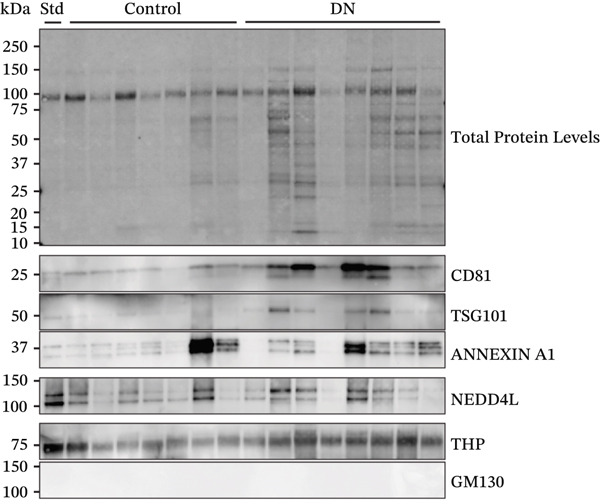
(a)

**Figure:**
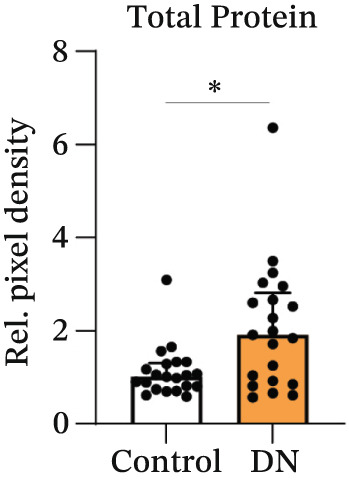
(b)

**Figure:**
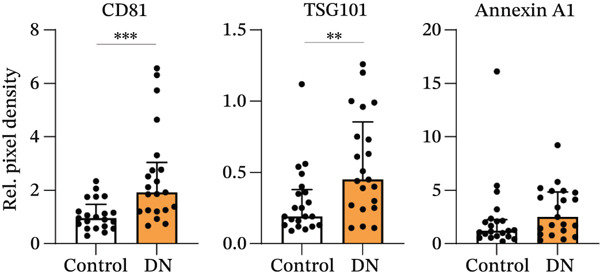
(c)

**Figure:**
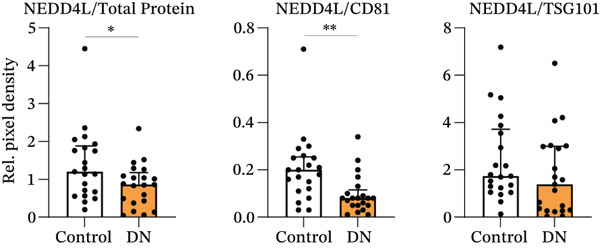
(d)

Concordance of EV markers was assessed for all samples (*n* = 42). There was a statistically significant, moderate‐to‐strong correlation of total protein with CD81 and TSG101, but no significant correlation between any of these markers and urinary creatinine or Annexin A1 (Table 2). Additionally, NEDD4L demonstrated statistically significant correlation with total protein, CD81 and TSG101, but not with urinary creatinine or Annexin A1 (Table 2). Therefore, total protein, CD81 and TSG101 were selected as EV markers for normalisation of NEDD4L in subsequent analysis.

**Table 2:** Pearson′s correlation matrix for urinary extracellular vesicle (EV) quantitative markers, urinary creatinine and NEDD4L levels in all participants, as detected through immunoblotting.

Variable	Mean	SD		1	2	3	4	5
1. Urinary creatinine (mg/dL)	82.92	47.20	Pearson correlation coefficient *r*	—				
			*p* value					
2. Normalised total protein magnitude (AU)	1.56	1.12	Pearson correlation coefficient *r*	0.25	—			
			*p* value	0.16				
3. Normalised CD81 magnitude (AU)	1.82	1.48	Pearson correlation coefficient *r*	0.18	**0.36** ^∗^	—		
			*p* value	0.25	**0.02**			
4. Normalised Annexin A1 magnitude (AU)	2.74	2.85	Pearson correlation coefficient *r*	−0.39	0.20	0.25	—	
			*p* value	0.81	0.21	0.11		
5. Normalised TSG101 magnitude (AU)	0.42	0.32	Pearson correlation coefficient *r*	0.16	**0.51** ^∗∗∗^	**0.42** ^∗∗^	0.09	—
			*p* value	0.31	**< 0.001**	**0.006**	0.56	
6. Normalised NEDD4L magnitude (AU)	0.53	0.56	Pearson correlation coefficient *r*	0.11	**0.77** ^∗∗∗^	**0.35** ^∗^	0.17	**0.38** ^∗^
			*p* value	0.49	**< 0.001**	**0.02**	0.29	**0.01**

*Note:* Correlation between EV markers, as well as between NEDD4L and EV markers was assessed in all samples using Pearson′s correlation coefficient. Normality was assumed by the central limit theorem. Total protein, CD81, Annexin A1, TSG101 and NEDD4L magnitudes were each normalised to a positive control on their respective blots. Spot urinary creatinine and normalised Annexin A1 demonstrated no statistically significant correlation with normalised total protein, normalised CD81, normalised TSG101, or with each other. Normalised total protein had a moderate correlation with normalised CD81 (*r* = 0.36; *p* = 0.02) and a strong correlation with normalised TSG101 (*r* = 0.51; *p* < 0.001). Normalised CD81 had a moderate correlation with TSG101 (*r* = 0.42; *p* = 0.006). Normalised NEDD4L had a strong correlation with normalised total protein (*r* = 0.77; *p* < 0.001), and moderate correlations with CD81 (*r* = 0.35; *p* = 0.02) and TSG101 (*r* = 0.38; *p* = 0.01). Bolded data highlight statistically significant values.

Abbreviations: AU, arbitrary units (noninterchangeable between factors); SD, standard deviation.

^∗^ *p* < 0.05.  ^∗∗^ *p* < 0.01.  ^∗∗∗^ *p* < 0.001.

### 3.4. Reduced NEDD4L Expression in Heterogeneous Urinary EVs From DN Patients

When normalised to total protein levels, NEDD4L expression was significantly reduced in DN samples (*p* = 0.038; median: 0.86; IQR: 0.78) as compared with controls without diabetes (median: 1.20; IQR: 1.27) (Figure 3d). Additionally, when normalised to CD81 levels, NEDD4L was also significantly reduced in DN samples (*p* = 0.002; median: 0.08; IQR: 0.07) compared with controls without diabetes (median: 0.20; IQR 0.14) (Figure 3d). When normalised to TSG101, there was no significant difference in NEDD4L expression between DN samples (*p* = 0.155; median: 1.38; IQR: 2.72) compared with control subjects without diabetes (median: 1.73; IQR: 2.57). Hence, despite the increased EV concentration observed in urine samples from DN patients, NEDD4L protein expression was significantly lower in urinary EVs from DN patients than in urinary EVs from control subjects.

## 4. Discussion

Herein, we report that the ubiquitin ligase NEDD4L is downregulated in patients with DN, as demonstrated in kidney biopsies and heterogeneous urinary EVs. These findings support the reduction of NEDD4L expression reported in a single‐cell transcriptomics study in the late distal convoluted tubule [24], as well as the results of our recent work showing reduced NEDD4‐2 expression in the kidneys of diabetic mice [25]. Thus, these findings suggest that this protein could serve as a novel biomarker for the detection of DN and progressive kidney function decline.

Currently, microalbuminuria is the most widely used indicator of DN, and is associated with progression of disease [36]. However, not all patients with DN exhibit microalbuminuria, highlighting the need for more sensitive biomarkers detectable in urine (due to the ease of urine collection). Importantly, the reduction in NEDD4L protein observed by immunofluorescence staining in the kidney biopsies from patients with T2DM and DN in this study (Figure 1) was also reflected in non‐invasive urine samples (Figure 3).

Recently, urinary EVs have gained popularity as a source of potential biomarkers in DN, with variations in total EV numbers and content, including changes in specific protein and mRNA concentrations linked to disease [37]. In the present study, we were able to isolate large numbers of heterogeneous EVs from urine samples and confirm their identity as lipid bilayer membrane–enclosed nanoparticles by TEM (Figure 2). We identified elevated amounts of heterogeneous EVs in urinary samples from DN patients. Urinary EV excretion has been shown to correlate with DN severity in another study conducted in patients with T2DM [38].

The increased intensity of EV protein markers on immunoblot in urinary samples from DN patients supports the increase in urinary EV concentration. Interestingly, it was predominantly small EVs within the size range of 100–200 nm that were elevated in DN, suggesting that these vesicles were primarily exosomes. The similar size distribution of urinary EVs from DN samples and controls without diabetes suggests that EV biogenesis was not affected, despite the increase in EV release. As urinary EVs are primarily of renal origin (mainly deriving from all parts of the nephrons and collecting ducts) [10], they are a reliable reflection of kidney pathology. This explains how our EV data support the findings observed in the kidney biopsy samples.

Our observation that total protein concentration is elevated in urinary EVs from DN patients (due to increased EV numbers) is supported by another study reporting significantly increased protein concentration of urinary EVs in patients with DN [39]. From the EV markers we assessed, exosomes derived from internal vesicles (CD81‐ and TSG101‐positive [40, 41]) were significantly elevated in DN, but not those budding directly from the plasma membrane (Annexin A1‐positive) [40], suggesting that these EVs are of a specific subtype and origin.

Interestingly, a reduction of NEDD4L in DN samples was observed when normalised to total protein or the exosomal marker CD81, but not TSG101. CD81 is a member of the tetraspanin protein family found on the cell surface and exosomes, whereas TSG101 protein is a core component of the endosomal sorting complex [42]. Hence, these differences may be due to the small sample size of the present study, or may reflect different cellular composition, such as variation in the proportion of cells that preferentially release CD81‐rich versus TSG101‐rich vesicles, or shifts in the relative abundance of different EV subpopulations. Importantly, the significant reduction of NEDD4L in DN samples may contribute to disease pathophysiology, or may be a protective mechanism to mitigate disease progression. In support of a causative role, deletion of NEDD4‐2 from the kidneys of mice leads to tubular epithelial cell apoptosis and CKD‐like pathology [20, 22]. Since NEDD4L is a critical regulator of several ion channels, reduced NEDD4L expression leads to elevated ion channel expression and enhanced sodium reabsorption in mice [19, 20]. Increased expression of ENaC and NCC has been reported in the distal nephron under diabetic conditions [43], and this can drive DN progression due to sodium retention and hypertension [44]. Although direct evidence for the regulation of ion channels such as ENaC and NCC in humans is lacking, polymorphisms in NEDD4L have been associated with increased salt sensitivity and risk of hypertension [45]. The elevated expression of these transporters may result from the reduced expression of NEDD4L. Hence, it would be interesting to assess the expression of ENaC and NCC in urinary EVs from patients with DN in future studies. In contrast, reduced expression of NEDD4L may restore levels of substrates that are reduced in DN. In support of this hypothesis, inhibition of NEDD4L by GDF‐15 (growth/differentiation factor 15, a member of the transforming growth factor‐*β* superfamily) has been shown to alleviate DN via a reduction of inhibitory‐*κ*B kinase (IKK) ubiquitination [46], suggesting that the downregulation of NEDD4L exerts a protective effect against DN. Further understanding of a causative or protective role of NEDD4L in DN requires additional mechanistic exploration.

The strengths of this work include the fact that this is the first study in humans to identify reduced NEDD4L expression in heterogeneous urinary EVs from patients with DN, within a larger and real‐world cohort as compared with many previous EV or DN biomarker studies. We acknowledge that the present study has several limitations. Firstly, given the pilot nature of this study, sample numbers were still relatively small, particularly for the kidney biopsy cohort. However, the reduced NEDD4L expression in the kidneys of DN patients was highly significant and warrants testing in larger samples in future studies. An additional limitation of the present study was the inability to test the discriminatory value of NEDD4L in EVs as a diagnostic test. However, our exploratory, pilot study was not designed or powered for this aspect, which would benefit from further investigation in larger validation studies. Secondly, a known limitation inherent to the exploratory, minimal‐risk nature of this pilot study conducted in an outpatient setting, was the risk of misclassification bias, since controls were identified through self‐declared health status only. Also, unlike cohort or case‐control studies that match groups to minimise confounding factors, the convenience sampling of controls resulted in different baseline characteristics between control and DN groups. We also did not collect baseline assessment of laboratory biochemical parameters (including eGFR) of control subjects. However, our findings justify larger‐scale investigation, such as matched comparison studies with serum biochemical measurements to exclude undiagnosed DN or other types of renal disease in control participants. Thirdly, 76.2% of the patients in the DN group (*n* = 16) had concurrent hypertension, whereas 28.6% of control subjects (*n* = 6) had mild‐to‐moderate hypertension. This reflects a real‐world scenario given the significant shared risk factors for T2DM and hypertension [47]. However, we cannot exclude a mixed aetiology of nephropathy related to both diabetes and hypertension in DN participants of our study. Hence, larger studies are needed to better stratify DN patients. Potential Type 1 error was mitigated by the inclusion of mild‐to‐moderate hypertension in the control group (28.6%), although this aspect may also influence the findings of our study. In future studies, inclusion of patients with hypertension (but without T2DM) as well as assessment of the effects of hypertension and antihypertensive drugs on NEDD4L expression levels would better delineate the differential contributions of diabetes and hypertension to the changes in NEDD4L expression among participants. Finally, we did not assess patients with T2DM and no evidence or clinical diagnosis of DN, to therefore ascertain the existence of differences in NEDD4L expression between patients with T2DM alone (without DN) and patients with T2DM and established DN. In Australia, this population is largely serviced in primary care settings rather than in tertiary care settings, and this may be a future area for investigation.

## 5. Conclusion

We have identified reduced NEDD4L expression levels in kidney biopsies and heterogeneous urinary EVs from patients with DN, as well as an increase in the total amount of urinary EVs in DN patients. The ease of urine collection and the small volume of sample size required highlight the potential suitability for clinical translation of this work. Established biomarkers for DN—such as serum creatinine and albuminuria—rely on serum and urinary markers that are not elevated until late stages of disease [48, 49]. The innovative use of urine to determine NEDD4L expression levels in urinary EVs provides a non‐invasive tool that paves the way for future larger studies aimed at determining whether this protein may serve as a useful biomarker to detect early pathological alterations and tubular injury prior to severe disease in both patients with T2DM who have established DN and those who do not have established DN. These studies would provide significant improvements for the early detection and prompt treatment of DN.

## Author Contributions

N.T., S.J. and J.A.M. conceptualized and designed the project; N.T. and J.A.M. performed experimental work; N.T. and J.A.M. analysed data and wrote the first manuscript draft; N.T., R.L.L., S.K., S.J. and J.A.M. edited the manuscript; S.K. procured funding, contributed intellectual input and provided laboratory resources. All authors discussed the results and commented on the manuscript.

## Funding

This study was supported by the National Health and Medical Research Council (10.13039/501100000925) (2007739) and Diabetes SA, Diabetes Australia (10.13039/501100000971) (Y23G‐MANJ).

## Conflicts of Interest

The authors declare no conflicts of interest.

## Supporting information


**Supporting Information** Additional supporting information can be found online in the Supporting Information section. Figure S1: Spot urinary creatinine levels. Figure S2: Formatted immunoblots of all samples collected (Set 2 and Set 3, that were not provided in main Figure 3). Figure S3: Complete uncropped immunoblots. Figure S4: Quantification of Tamm–Horsfall protein (THP) and extracellular vesicle (EV) markers.

## Data Availability

All data are presented in the main manuscript and additional supporting information.

## References

[1] Duncan B. B. , Magliano D. J. , and Boyko E. J. , IDF Diabetes Atlas 11th Edition 2025: Global Prevalence and Projections for 2050, Nephrology, Dialysis, Transplantation. (2025) 41, no. 1, 7–9, 10.1093/ndt/gfaf177, 40874767. 40874767

[2] IDF (International Diabetes Federation) , Diabetes and Kidney Disease, 2023, accessed on December 27, 2025, https://diabetesatlas.org/resources/idf-diabetes-atlas-reports/diabetes-and-kidney-disease/.

[3] ANZDATA Registry , 48th Report, Chapter 1: Incidence of Kidney Failure With Replacement Therapy, accessed on 27 December, 2025, https://anzorrg.org.au/reports/anzdata-48th-annual-report-2025-data-survey-2024.

[4] GBD Kidney Failure With Replacement Therapy Collaborators , Global, Regional, and National Prevalence of Kidney Failure With Replacement Therapy and Associated Aetiologies, 1990-2023: A Systematic Analysis for the Global Burden of Disease Study 2023, Lancet Global Health. (2025) 13, no. 8, e1378–e1395, 10.1016/S2214-109X(25)00198-6, 40712611. 40712611

[5] Lim W. H. , Johnson D. W. , McDonald S. P. , Hawley C. , Clayton P. A. , Jose M. D. , and Wong G. , Impending Challenges of the Burden of End-Stage Kidney Disease in Australia, Medical Journal of Australia. (2019) 211, no. 8, 374–380 e373, 10.5694/mja2.50354, 2-s2.0-85073632021. 31595516

[6] World Health Organization , Global Report on Diabetes, 2016, accessed on 27 December, 2025, https://www.who.int/publications/i/item/9789241565257.

[7] Habib S. L. , Diabetes and Renal Tubular Cell Apoptosis, World Journal of Diabetes. (2013) 4, no. 2, 27–30, 10.4239/wjd.v4.i2.27, 23593533. 23593533 PMC3627416

[8] Kidney Health Australia , Chronic Kidney Disease (CKD) Management in Primary Care 5th Edition, 2024, accessed on 31 December, 2025, https://kidney.org.au/wp-content/uploads/2025/11/KHA-CKD-Handbook-5th-Ed-July2024.pdf.

[9] Merchant M. L. , Rood I. M. , Deegens J. K. J. , and Klein J. B. , Isolation and Characterization of Urinary Extracellular Vesicles: Implications for Biomarker Discovery, Nature Reviews Nephrology. (2017) 13, no. 12, 731–749, 10.1038/nrneph.2017.148, 2-s2.0-85034028641, 29081510. 29081510 PMC5941934

[10] Lu Y. , Liu D. , Feng Q. , and Liu Z. , Diabetic Nephropathy: Perspective on Extracellular Vesicles, Frontiers in Immunology. (2020) 11, 10.3389/fimmu.2020.00943. PMC7283536 32582146

[11] Grange C. and Bussolati B. , Extracellular Vesicles in Kidney Disease, Nature Reviews Nephrology. (2022) 18, no. 8, 499–513, 10.1038/s41581-022-00586-9, 35641620. 35641620 PMC9152665

[12] Hill N. , Michell D. L. , Ramirez-Solano M. , Sheng Q. , Pusey C. , Vickers K. C. , and Woollard K. J. , Glomerular Endothelial Derived Vesicles Mediate Podocyte Dysfunction: A Potential Role for miRNA, PLoS One. (2020) 15, no. 3, e0224852, 10.1371/journal.pone.0224852. 32214346 PMC7098579

[13] Barreiro K. , Dwivedi O. P. , Leparc G. , Rolser M. , Delic D. , Forsblom C. , Groop P. H. , Groop L. , Huber T. B. , Puhka M. , and Holthofer H. , Comparison of Urinary Extracellular Vesicle Isolation Methods for Transcriptomic Biomarker Research in Diabetic Kidney Disease, Journal of Extracellular Vesicles. (2020) 10, no. 2, e12038, 10.1002/jev2.12038. 33437407 PMC7789228

[14] Leung P. Y. M. , Katerelos M. , Choy S. , Cook N. , Lee M. , Paizis K. , Abboud A. , Manning J. A. , Mount P. F. , and Power D. A. , Expression of NEDD4L and ENaC in Urinary Extracellular Vesicles in Pre-Eclampsia, Hypertension in Pregnancy. (2023) 42, no. 1, 2232029, 10.1080/10641955.2023.2232029. 37417251

[15] Manning J. A. , Henshall T. L. , and Kumar S. , NEDD4-2-Dependent Control of Na(+) Homeostasis and Renal Disease, Cell Cycle. (2018) 17, no. 1, 1–2, 10.1080/15384101.2017.1386514, 2-s2.0-85039054745, 29108451. 29108451 PMC5815436

[16] Goel P. , Manning J. A. , and Kumar S. , NEDD4-2 (NEDD4L): The Ubiquitin Ligase for Multiple Membrane Proteins, Gene. (2015) 557, no. 1, 1–10, 10.1016/j.gene.2014.11.051, 2-s2.0-84920085136, 25433090. 25433090 PMC6636357

[17] Manning J. A. and Kumar S. , Physiological Functions of Nedd4-2: Lessons From Knockout Mouse Models, Trends in Biochemical Sciences. (2018) 43, no. 8, 635–647, 10.1016/j.tibs.2018.06.004, 2-s2.0-85049319868, 30056838. 30056838

[18] Dieter M. , Palmada M. , Rajamanickam J. , Aydin A. , Busjahn A. , Boehmer C. , Luft F. C. , and Lang F. , Regulation of Glucose Transporter SGLT1 by Ubiquitin Ligase Nedd4-2 and Kinases SGK1, SGK3, and PKB, Obesity Research. (2004) 12, no. 5, 862–870, 15166308. 15166308 10.1038/oby.2004.104

[19] Ronzaud C. , Loffing-Cueni D. , Hausel P. , Debonneville A. , Malsure S. R. , Fowler-Jaeger N. , Boase N. A. , Perrier R. , Maillard M. , Yang B. , Stokes J. B. , Koesters R. , Kumar S. , Hummler E. , Loffing J. , and Staub O. , Renal Tubular NEDD4-2 Deficiency Causes NCC-Mediated Salt-Dependent Hypertension, Journal of Clinical Investigation. (2013) 123, no. 2, 657–665, 10.1172/JCI61110, 2-s2.0-84873350794, 23348737. 23348737 PMC3561795

[20] Henshall T. L. , Manning J. A. , Alfassy O. S. , Goel P. , Boase N. A. , Kawabe H. , and Kumar S. , Deletion of Nedd4-2 Results in Progressive Kidney Disease in Mice, Cell Death and Differentiation. (2017) 24, no. 12, 2150–2160, 10.1038/cdd.2017.137, 2-s2.0-85034089520, 28862701. 28862701 PMC5686353

[21] Manning J. A. , Shah S. S. , Henshall T. L. , Nikolic A. , Finnie J. , and Kumar S. , Dietary Sodium Modulates Nephropathy in Nedd4-2-Deficient Mice, Cell Death and Differentiation. (2020) 27, no. 6, 1832–1843, 10.1038/s41418-019-0468-5, 31802037. 31802037 PMC7244563

[22] Manning J. A. , Shah S. S. , Nikolic A. , Henshall T. L. , Khew-Goodall Y. , and Kumar S. , The Ubiquitin Ligase NEDD4-2/NEDD4L Regulates Both Sodium Homeostasis and Fibrotic Signaling to Prevent End-Stage Renal Disease, Cell Death & Disease. (2021) 12, no. 4, 10.1038/s41419-021-03688-7. PMC8046789 33854040

[23] Hasstedt S. J. , Highland H. M. , Elbein S. C. , Hanis C. L. , Das S. K. , and American Diabetes Association GENNID Study Group , Five Linkage Regions Each Harbor Multiple Type 2 Diabetes Genes in the African American Subset of the GENNID Study, Journal of Human Genetics. (2013) 58, no. 6, 378–383, 10.1038/jhg.2013.21, 2-s2.0-84880913229, 23552671. 23552671 PMC3692593

[24] Wilson P. C. , Wu H. , Kirita Y. , Uchimura K. , Ledru N. , Rennke H. G. , Welling P. A. , Waikar S. S. , and Humphreys B. D. , The Single-Cell Transcriptomic Landscape of Early Human Diabetic Nephropathy, Proceedings of the National Academy of Sciences of the United States of America. (2019) 116, no. 39, 19619–19625, 10.1073/pnas.1908706116, 2-s2.0-85072630804, 31506348. 31506348 PMC6765272

[25] Manning J. A. , Jesudason S. , Moretti P. A. B. , Pitson S. M. , Chou A. S. Y. , Shabbar M. , Saad S. , Pollock C. , and Kumar S. , *Nedd4-2* Ablation in Kidney Improves Glycaemic Control in Diabetic Mice, Cell Death & Disease. (2025) 16, no. 1, 10.1038/s41419-025-07826-3. PMC12228766 40617804

[26] Farooq A. U. , Gembus K. , Sandow J. J. , Webb A. , Mathivanan S. , Manning J. A. , Shah S. S. , Foot N. J. , and Kumar S. , K-29 Linked Ubiquitination of Arrdc4 Regulates Its Function in Extracellular Vesicle Biogenesis, Journal of Extracellular Vesicles. (2022) 11, no. 2, e12188, 10.1002/jev2.12188. 35106941 PMC8807422

[27] Konstas A. A. , Shearwin-Whyatt L. M. , Fotia A. B. , Degger B. , Riccardi D. , Cook D. I. , Korbmacher C. , and Kumar S. , Regulation of the Epithelial Sodium Channel by N4WBP5A, a Novel Nedd4/Nedd4-2-Interacting Protein, Journal of Biological Chemistry. (2002) 277, no. 33, 29406–29416, 10.1074/jbc.M203018200, 2-s2.0-0037119459, 12050153. 12050153

[28] Kidney Disease: Improving Global Outcomes (KDIGO) CKD Work Group , Clinical Practice Guideline for the Evaluation and Management of Chronic Kidney Disease, Kidney International. (2024) 105, no. Supplement 4, S117–S314, accessed on December 4, 2025, https://kdigo.org/wp-content/uploads/2024/03/KDIGO-2024-CKD-Guideline.pdf. 38490803 10.1016/j.kint.2023.10.018

[29] Inker L. A. , Eneanya N. D. , Coresh J. , Tighiouart H. , Wang D. , Sang Y. , Crews D. C. , Doria A. , Estrella M. M. , Froissart M. , Grams M. E. , Greene T. , Grubb A. , Gudnason V. , Gutierrez O. M. , Kalil R. , Karger A. B. , Mauer M. , Navis G. , Nelson R. G. , Poggio E. D. , Rodby R. , Rossing P. , Rule A. D. , Selvin E. , Seegmiller J. C. , Shlipak M. G. , Torres V. E. , Yang W. , Ballew S. H. , Couture S. J. , Powe N. R. , Levey A. S. , and Chronic Kidney Disease Epidemiology Collaboration , New Creatinine- and Cystatin C-Based Equations to Estimate GFR Without Race, New England Journal of Medicine. (2021) 385, no. 19, 1737–1749, 10.1056/NEJMoa2102953, 34554658. 34554658 PMC8822996

[30] Lu S. , Robyak K. , and Zhu Y. , The CKD-EPI 2021 Equation and Other Creatinine-Based Race-Independent eGFR Equations in Chronic Kidney Disease Diagnosis and Staging, Journal of Applied Laboratory Medicine. (2023) 8, no. 5, 952–961, 10.1093/jalm/jfad047, 37534520. 37534520

[31] Jones D. W. , Ferdinand K. C. , Taler S. J. , Johnson H. M. , Shimbo D. , Abdalla M. , Altieri M. M. , Bansal N. , Bello N. A. , Bress A. P. , Carter J. , Cohen J. B. , Collins K. J. , Commodore-Mensah Y. , Davis L. L. , Egan B. , Khan S. S. , Lloyd-Jones D. M. , Melnyk B. M. , Mistry E. A. , Ogunniyi M. O. , Schott S. L. , Smith S. C. , Talbot A. W. , Vongpatanasin W. , Watson K. E. , Whelton P. K. , and Williamson J. D. , 2025 AHA/ACC/AANP/AAPA/ABC/ACCP/ACPM/AGS/AMA/ASPC/NMA/PCNA/SGIM Guideline for the Prevention, Detection, Evaluation and Management of High Blood Pressure in Adults: A Report of the American College of Cardiology/American Heart Association Joint Committee on Clinical Practice Guidelines, Hypertension. (2025) 82, no. 10, e212–e316, 10.1161/HYP.0000000000000249, 40811516. 40811516

[32] Gauthier S. , Pranke I. , Jung V. , Martignetti L. , Stoven V. , Nguyen-Khoa T. , Semeraro M. , Hinzpeter A. , Edelman A. , Guerrera I. C. , and Sermet-Gaudelus I. , Urinary Exosomes of Patients With Cystic Fibrosis Unravel CFTR-Related Renal Disease, International Journal of Molecular Sciences. (2020) 21, no. 18, 6625, 10.3390/ijms21186625. 32927759 PMC7554933

[33] Holthofer H. , Schulte B. A. , and Spicer S. S. , Expression of Binding Sites for *Dolichos Biflorus* Agglutinin at the Apical Aspect of Collecting Duct Cells in Rat Kidney, Cell and Tissue Research. (1987) 249, no. 3, 481–485, 10.1007/BF00217319, 2-s2.0-0023404524, 2822251. 2822251

[34] Li Y. , Wen X. , and Liu Y. , Tubular Cell Dedifferentiation and Peritubular Inflammation Are Coupled by the Transcription Regulator Id1 in Renal Fibrogenesis, Kidney International. (2012) 81, no. 9, 880–891, 10.1038/ki.2011.469, 2-s2.0-84862784957, 22278018. 22278018 PMC3326205

[35] Wachalska M. , Koppers-Lalic D. , van Eijndhoven M. , Pegtel M. , Geldof A. A. , Lipinska A. D. , van Moorselaar R. J. , and Bijnsdorp I. V. , Protein Complexes in Urine Interfere With Extracellular Vesicle Biomarker Studies, Journal of Circulating Biomarkers. (2016) 5, 10.5772/62579, 2-s2.0-85021139694. PMC5548314 28936252

[36] Lee S. Y. and Choi M. E. , Urinary Biomarkers for Early Diabetic Nephropathy: Beyond Albuminuria, Pediatric Nephrology. (2015) 30, no. 7, 1063–1075, 10.1007/s00467-014-2888-2, 2-s2.0-84929962114, 25060761. 25060761 PMC4305495

[37] Saenz-Pipaon G. , Echeverria S. , Orbe J. , and Roncal C. , Urinary Extracellular Vesicles for Diabetic Kidney Disease Diagnosis, Journal of Clinical Medicine. (2021) 10, no. 10, 2046, 10.3390/jcm10102046. 34064661 PMC8151759

[38] De S. , Kuwahara S. , Hosojima M. , Ishikawa T. , Kaseda R. , Sarkar P. , Yoshioka Y. , Kabasawa H. , Iida T. , Goto S. , Toba K. , Higuchi Y. , Suzuki Y. , Hara M. , Kurosawa H. , Narita I. , Hirayama Y. , Ochiya T. , and Saito A. , Exocytosis-Mediated Urinary Full-Length Megalin Excretion Is Linked With the Pathogenesis of Diabetic Nephropathy, Diabetes. (2017) 66, no. 5, 1391–1404, 10.2337/db16-1031, 2-s2.0-85019350898, 28289043. 28289043

[39] Gu D. , Chen Y. , Masucci M. , Xiong C. , Zou H. , and Holthofer H. , Potential Urine Biomarkers for the Diagnosis of Prediabetes and Early Diabetic Nephropathy Based on ISN CKHDP Program, Clinical Nephrology. (2020) 93, no. 1, 129–133, 10.5414/CNP92S123, 32145759. 32145759

[40] Jeppesen D. K. , Fenix A. M. , Franklin J. L. , Higginbotham J. N. , Zhang Q. , Zimmerman L. J. , Liebler D. C. , Ping J. , Liu Q. , Evans R. , Fissell W. H. , Patton J. G. , Rome L. H. , Burnette D. T. , and Coffey R. J. , Reassessment of Exosome Composition, Cell. (2019) 177, no. 2, 428–445.e18, 10.1016/j.cell.2019.02.029, 2-s2.0-85063038079. 30951670 PMC6664447

[41] Teng F. and Fussenegger M. , Shedding Light on Extracellular Vesicle Biogenesis and Bioengineering, Advanced Science. (2020) 8, no. 1, 2003505, 10.1002/advs.202003505, 33437589. 33437589 PMC7788585

[42] Gurung S. , Perocheau D. , Touramanidou L. , and Baruteau J. , The Exosome Journey: From Biogenesis to Uptake and Intracellular Signalling, Cell Communication and Signaling: CCS. (2021) 19, no. 1, 10.1186/s12964-021-00730-1. PMC8063428 33892745

[43] Spires D. , Manis A. D. , and Staruschenko A. , Ion Channels and Transporters in Diabetic Kidney Disease, Current Topics in Membranes. (2019) 83, 353–396, 10.1016/bs.ctm.2019.01.001, 2-s2.0-85061645281, 31196609. 31196609 PMC6815098

[44] Pearce D. , Soundararajan R. , Trimpert C. , Kashlan O. B. , Deen P. M. , and Kohan D. E. , Collecting Duct Principal Cell Transport Processes and Their Regulation, Clinical Journal of the American Society of Nephrology. (2015) 10, no. 1, 135–146, 10.2215/CJN.05760513, 2-s2.0-84922618496, 24875192. 24875192 PMC4284417

[45] Dahlberg J. , Nilsson L. O. , von Wowern F. , and Melander O. , Polymorphism in NEDD4L Is Associated With Increased Salt Sensitivity, Reduced Levels of P-Renin and Increased Levels of Nt-proANP, PLoS One. (2007) 2, no. 5, e432, 10.1371/journal.pone.0000432, 2-s2.0-39749189309, 17487281. 17487281 PMC1855992

[46] Zhang X. , Wang S. , Chong N. , Chen D. , Shu J. , Sun J. , Sun Z. , Wang R. , Wang Q. , and Xu Y. , GDF-15 Alleviates Diabetic Nephropathy via Inhibiting NEDD4L-Mediated IKK/NF-*κ*B Signalling Pathways, International Immunopharmacology. (2024) 128, 111427, 10.1016/j.intimp.2023.111427, 38181673. 38181673

[47] Cheung B. M. and Li C. , Diabetes and Hypertension: Is there a Common Metabolic Pathway?, Current Atherosclerosis Reports. (2012) 14, no. 2, 160–166, 10.1007/s11883-012-0227-2, 2-s2.0-84862825466, 22281657. 22281657 PMC3314178

[48] Wani Z. A. , Ahmed S. , Saleh A. , Anna V. R. , Fahelelbom K. M. , Raju S. K. , Abu-Rayyan A. , and Bhat A. R. , Biomarkers in Diabetic Nephropathy: A Comprehensive Review of Their Role in Early Detection and Disease Progression Monitoring, Diabetes Research and Clinical Practice. (2025) 226, 112292, 10.1016/j.diabres.2025.112292, 40466742. 40466742

[49] Karimi F. , Moazamfard M. , Taghvaeefar R. , Sohrabipour S. , Dehghani A. , Azizi R. , and Dinarvand N. , Early Detection of Diabetic Nephropathy Based on Urinary and Serum Biomarkers: An Updated Systematic Review, Advanced Biomedical Research. (2024) 13. 10.4103/abr.abr_461_23 PMC11665175 39717256

