# Supplementary material for: Increased Urinary Extracellular Vesicles and Reduced Expression of NEDD4L in Patients With Type 2 Diabetes and Diabetic Nephropathy
Source: J Diabetes Res. 2026 Mar 9;2026:3850490. doi: 10.1155/jdr/3850490 (PMC12968427; doi:10.1155/jdr/3850490)
Supplement: Supplementary file 1 — Supporting Information Additional supporting information can be found online in the Supporting Information section. Figure S1: Spot urinary creatinine levels. Figure S2: Formatted immunoblots of all samples collected (Set 2 and Set 3, that were not provided in main Figure 3). Figure S3: Complete uncropped immunoblots. Figure S4: Quantification of Tamm–Horsfall protein (THP) and extracellular vesicle (EV) markers. [file JDR-2026-3850490-s001.pdf]

**Supplementary Material**

The following figures are provided in this PDF.

**Supplementary Figure S1.** Spot urinary creatinine levels.

**Supplementary Figure S2.** Formatted immunoblots of all samples collected (Set 2 and Set 3, that were not provided in main Figure 3).

**Supplementary Figure S3.** Complete uncropped immunoblots.

**Supplementary Figure S4.** Quantification of Tamm-Horsfall Protein (THP) and extracellular vesicle (EV) markers.

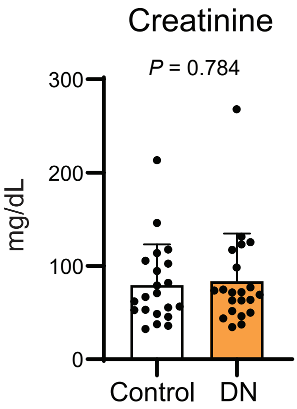


**Supplementary Figure S1.** Spot urinary creatinine levels. Creatinine was measured in an aliquot of each urine sample prior to extracellular vesicle (EV) isolation (n = 21 for each group). Data are presented as mean ± standard error of the mean (SEM). No significant differences were observed between control and DN samples (Mann-Whitney U test).

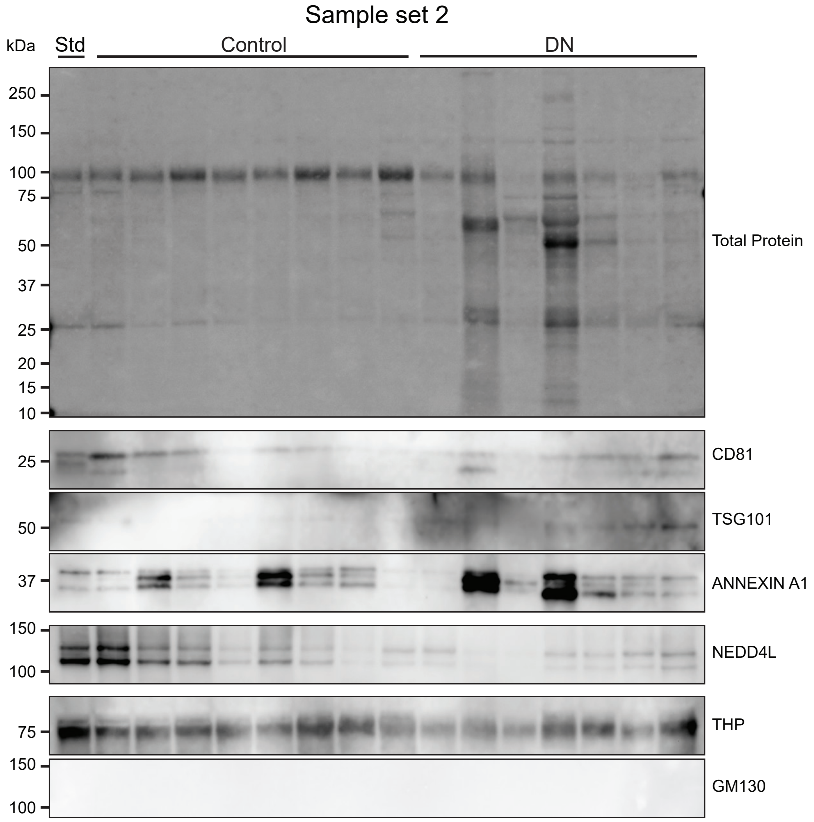


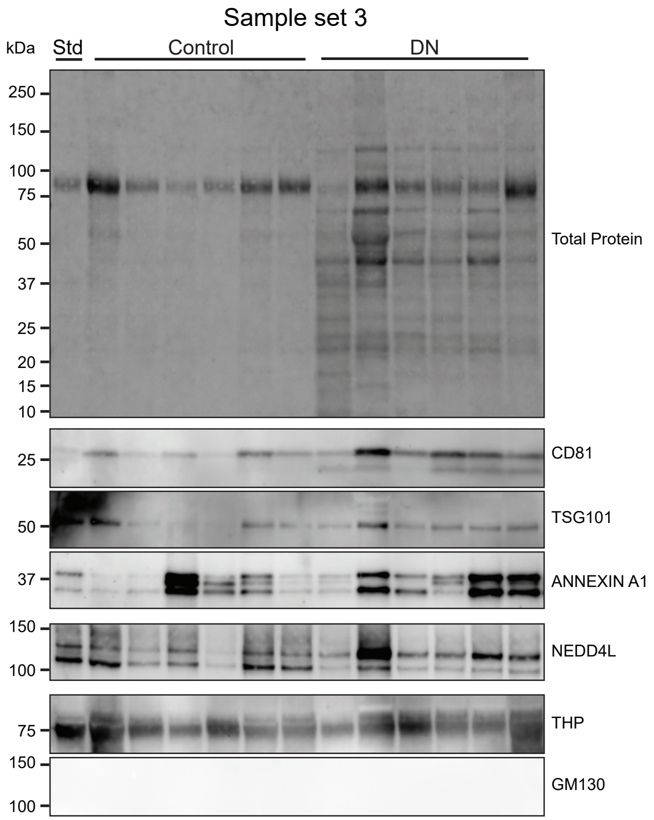


**Supplementary Figure S2.** Formatted immunoblots of all samples collected (Set 2 and Set 3, that were not provided in main Figure 3). Membranes were assessed for total protein levels, and then probed for specific proteins. All participant samples across three membranes were normalised to a standard in lane 1.

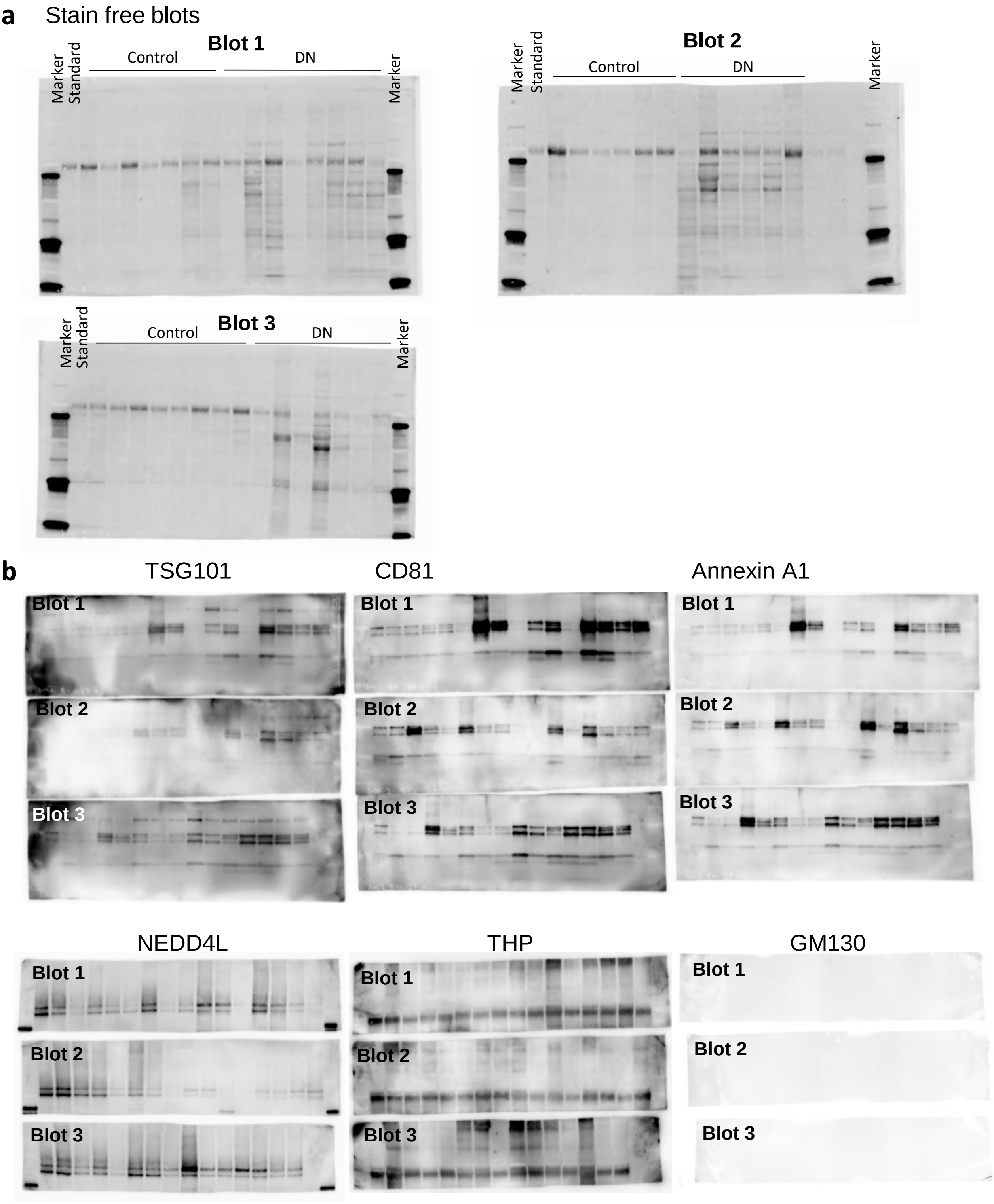


**Supplementary Figure S3**. Complete uncropped immunoblots. Blots were cut in half prior to probing. Each blot starts and ends with Kaleidoscope marker lane. A common standard (Lane 2; duplicate JM1 aliquot divided into thirds) was loaded onto all blots to enable normalization across experiments. Each blot has control samples and DN samples. Blot 2 has two samples not used, collected for other purposes.

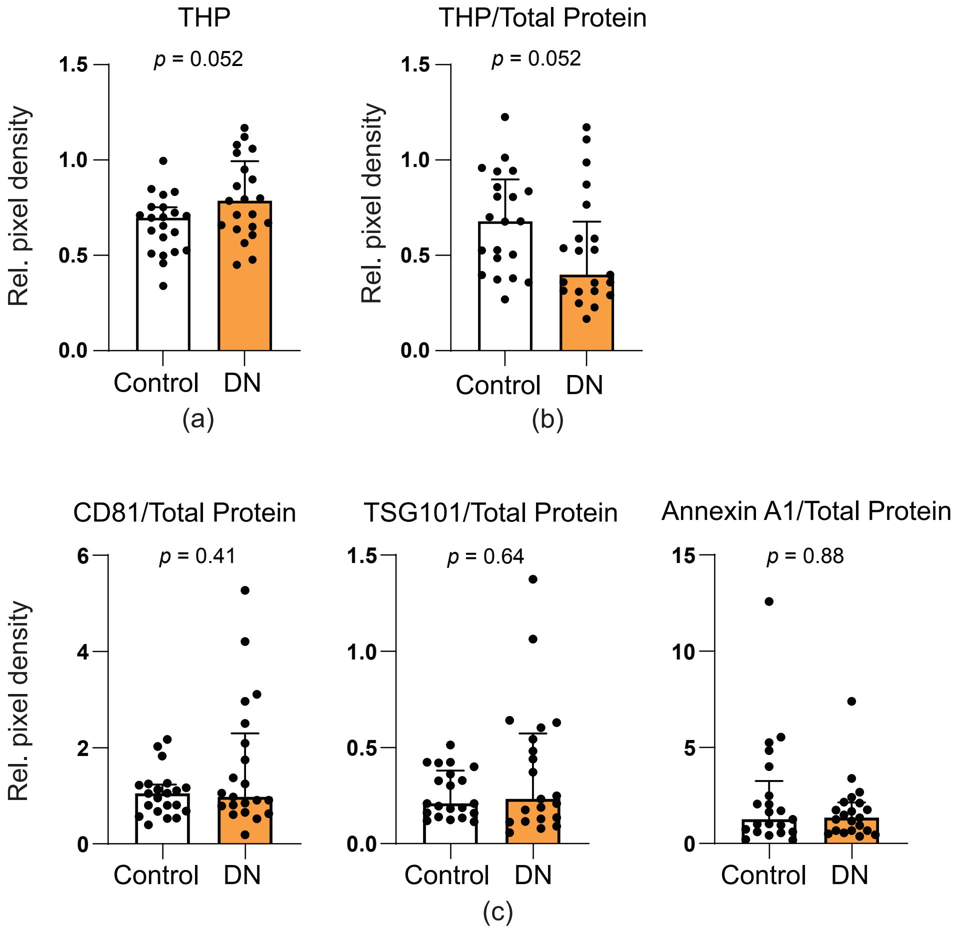


**Supplementary Figure S4.** Quantification of Tamm-Horsfall Protein (THP) and extracellular vesicle (EV) markers. (a) THP normalised to standard. (b) THP normalised to total protein. (c) CD81, TSG101 and Annexin A1 normalised to total protein. Data presented from n = 21, as median with IQR (Mann Whitney U Test). No significant differences between Control and DN samples were observed.
